# Dietary Intake and Oral Glucose Tolerance Test Results in Women with Gestational Diabetes

**DOI:** 10.3390/jcm13102948

**Published:** 2024-05-16

**Authors:** Lucas Almeida das Chagas, Maria Regina Torloni, Luiz Gonzaga Ribeiro Silva-Neto, Patricia Medici Dualib, Rosângela Maria Lopes de Sousa, Jalila Andréa Sampaio Bittencourt, Edward Araujo Júnior, Roberta Granese, Rosiane Mattar

**Affiliations:** 1 Department of Obstetrics, Paulista School of Medicine, Federal University of São Paulo (EPM-UNIFESP), São Paulo 04023-062, SP, Brazil; lucaschagas94@hotmail.com (L.A.d.C.); torlonimr@gmail.com (M.R.T.); araujojred@terra.com.br (E.A.J.); rosiane.mattar@unifesp.br (R.M.); 2 Evidence Based Health Care, Department of Medicine, Paulista School of Medicine, Federal University of São Paulo (EPM-UNIFESP), São Paulo 04023-900, SP, Brazil; 3 Department of Nutrition, Paulista School of Medicine, Federal University of São Paulo (EPM-UNIFESP), São Paulo 04023-062, SP, Brazil; lgrs.neto@unifesp.br; 4 Discipline of Endocrinology, Department of Medicine, Paulista School of Medicine, Federal University of São Paulo (EPM-UNIFESP), São Paulo 04038-001, SP, Brazil; patricia.dualib@uol.com.br; 5 Department of Nutrition, CEUMA University, São Luiz 65075-120, MA, Brazil; coordenacao.nutricao-slz@ceuma.br; 6 Laboratory of Biological Information Processing, Department of Electrical Engineering, Federal University of Maranhão (UFMA), São Luiz 65080-805, MA, Brazil; jalilabittencourt@gmail.com; 7 Department of Biomedical and Dental Sciences and Morphofunctional Imaging, “G. Martino” University Hospital, 98100 Messina, Italy

**Keywords:** gestational diabetes mellitus, diet, glucose tolerance test

## Abstract

**Background/Objective:** Diet is a risk factor for gestational diabetes mellitus (GDM). There are few studies on women’s diet and glucose tolerance test (GTT) results during pregnancy. The objective of this study was to evaluate the relationship between one’s previous diet and the number of abnormal values on the diagnostic GTT in women with GDM. We hypothesized that there would be an inverse relation between antioxidant micronutrient consumption and the number of abnormal GTT values. **Methods:** This cross-sectional study included 60 women diagnosed with GDM (2-h, 75 g-GTT), divided in two groups as follows: 1 abnormal glucose value and 2–3 abnormal values. Shortly after the diagnosis, participants answered a validated food frequency questionnaire to assess their food consumption in the last 6 months. The Mann–Whitney test was used to compare the dietary intake of the participants in the two groups. **Results:** The participant characteristics were similar. The median intake of total calories, carbohydrates, lipids, and proteins did not differ significantly between groups. Participants with 1 abnormal GTT value had significantly higher intakes of fiber (11.9 vs. 11.0 g/day *p* = 0.049), vitamin D (40.6 vs. 40.4 mcg/day *p* = 0.049), and vitamin C (180.0 vs. 151.0 mg/day *p* = 0.008) than those with 2–3 abnormal values. **Conclusions:** Our results suggest a possible association between the consumption of fiber and antioxidant micronutrients and the number of abnormal GTT values.

## 1. Introduction

Gestational diabetes mellitus (GDM) is defined as glucose intolerance of variable severity, with the onset or first recognition occurring during pregnancy, which does not meet the diagnostic criteria for pre-existing diabetes, and may or may not persist after delivery [1]. Over the course of normal pregnancy, maternal and placental hormones induce a physiologic insulin resistance to ensure that the fetus receives a constant and adequate amount of glucose. Normal women respond to this by increasing the pancreatic production of insulin to maintain glucose homeostasis. Women whose pancreatic response is insufficient to overcome this pregnancy-induced insulin resistance develop GDM [2]. GDM is one of the most common complications of pregnancy, with a worldwide prevalence between 8.4% and 30.9% [3,4]. This variation is due to differences in population characteristics (such as age and obesity) and diagnostic criteria [3,4]. In Brazil, the prevalence of GDM is 18% according to the criteria proposed by the International Association of Diabetes and Pregnancy Study Group [5]. Rates of GDM are increasing, in parallel with the increasing rates of obesity, advanced maternal age, and unhealthy lifestyle habits across most populations [6,7,8].

GDM increases the risks of obstetric and perinatal complications (e.g., gestational hypertension, neonatal hypoglycemia, macrosomia) [9], the later development of type 2 diabetes in the mother, and abnormal glucose metabolism in exposed offspring [10]. However, timely and appropriate GDM treatment can decrease the risks of these complications [11,12]. Due to its high prevalence and negative impact on maternal and child health, GDM is an important public health issue.

According to recommendations from the World Health Organization (WHO), the diagnosis of GDM should be made in pregnant women who have ≥1 abnormal glucose values on a 75 g oral glucose tolerance test (GTT), which consists of measuring the serum glucose levels at three time points (fasting, 1 h, and 2 h after the glucose load) [1]. A larger number of abnormal glucose values on the GTT has been associated with higher risks of adverse maternal and perinatal outcomes and the need of antenatal insulin treatment for glycemic control [13,14,15,16]. Investigators suggest that the glucose metabolism and insulin sensitivity of women with >1 abnormal glucose value on the GTT are more compromised than those of women with only 1 abnormal value, characterizing a more severe subtype of GDM [13,14,15,16].

There is ample evidence suggesting that changing eating habits before and at the beginning of pregnancy can reduce the risk of developing GDM [17,18,19,20,21,22]. However, there are fewer studies on the effects of diet on the severity of GDM, and we could not identify any Brazilian study concerning this topic. A large trial conducted in Spain showed that a nutritional intervention in early pregnancy, consisting of a Mediterranean diet with added extra virgin olive oil and nuts, reduced the incidence of GDM in 30%, and significantly fewer women who developed GDM required insulin therapy, suggesting that women in the intervention group developed a milder form of the disease [23,24,25]. Adopting a diet which is rich in dietary fiber and antioxidants, with a greater variety of fruits and vegetables, could minimize the effect of oxidative stress, thus helping to prevent and control GDM and its adverse effects [26,27]. Oxidative stress is the result of an imbalance between the production and detoxication of free radicals, which are highly reactive molecules that include reactive oxygen species (ROS). The excessive production of ROS can induce inflammatory responses and cause direct tissular damage and cell death, especially in mitochondria-rich tissues, such as pancreatic Beta cells, thus reducing insulin production [28]. Therefore, since oxidative stress is involved in the pathogenesis of GDM, diets rich in antioxidants could modulate this effect.

The objective of this study was to evaluate the relationship between the type of diet consumed before the diagnosis of GDM and the number of abnormal glucose values on the GTT, an indicator of disease severity. Our hypothesis was that women with a larger number of abnormal glucose values on the GTT would exhibit worse diet quality (poorer in antioxidants and fibers) than those with fewer abnormal values.

## 2. Methods

### 2.1. Study Design and Period

This is a secondary analysis of a case–control study carried out at the Federal University of São Paulo (UNIFESP), a public institution located in São Paulo, Brazil, in 2018–2019, that evaluated the food consumption of women with GDM with and without hepatic steatosis [29]. The study was conducted according to the guidelines of the Declaration of Helsinki, and was approved by the institutional review board of Federal University of São Paulo (protocol code CAEE 86210318.0.0000.5505, 16 May 2018). All participants signed an informed consent form at the time of recruitment.

### 2.2. Participants

All pregnant women managed at UNIFESP´s antenatal care clinics routinely perform a fasting blood glucose test in the first trimester of pregnancy. All those with normal fasting blood glucose (<92 mg/dL) undergo a 75 g 2 h GTT at 24–32 weeks of gestation. Women with any of the following receive a diagnosis of GDM: fasting 92–125 mg/dL, 1 h ≥ 180 mg/dL, or 2 h 153–199 mg/dL [1]. Women who are diagnosed with GDM are referred to UNIFESP´s Diabetes Center, a free public outpatient clinic where they continue prenatal care with a specialized multiprofessional team which includes obstetrician–gynecologists, nurses, endocrinologists, and nutritionists. At their first visit to the Diabetes Center, before they have had any contact with other healthcare professionals, all women with a recent diagnosis of GDM were approached by the first author, who invited them to participate in the study. Women who were 18–45 years of age, with a live singleton pregnancy between 26–34 weeks, having received a diagnosis of GDM in the last 2 weeks, and not yet undergoing any GDM treatment (including nutritional counselling), were eligible to participate. The exclusion criteria included the following: illiteracy, having a mental, hearing, or visual disability that made data collection impossible, foreigners who were not fluent in Portuguese, women with bariatric surgery, infectious diseases that can affect eating habits (such as tuberculosis), women who were on any type of diet, with a history or current alcohol consumption > 20 g/day, type 1 or 2 diabetes, ascites, a history or current diagnosis of liver diseases (including hepatitis, cirrhosis, alcoholic fatty liver disease, or malignant tumors), use of medications potentially associated with hepatic steatosis (including tamoxifen, estrogen, or diltiazem) in the last six months, and women who could not remember their weight before pregnancy. We also excluded from the study women who did not complete the food frequency questionnaire for any reason, including the inability to recall their food consumption, portions, or frequency in the last six months. For this study, we categorized participants into two groups, based on the results of their GTT, as follows: those with only 1 abnormal value and those with 2–3 abnormal values.

### 2.3. Data Collection

Immediately after recruitment, all eligible women underwent an abdominal ultrasound (US) scan at the Diabetes Center. The same physician used portable equipment (Sanoace Pico, Medison, Seoul, Republic of Korea) to perform all exams. He classified each woman as having a normal liver, or as having mild, moderate, or severe diffuse fatty infiltration [29,30]. The principal investigator then weighed and measured each participant using an electronic digital scale (MEA 07700, Plenna, Sao Paulo, Brazil) and a stadiometer. The investigator then interviewed women to collect sociodemographic data, family and obstetric history, and information regarding pre-pregnancy weight. Women were also asked about the duration and frequency of regular physical activity in the six months before getting pregnant; those who reported at least 30 min of regular physical activity/week were considered physically active [31]. Participants then completed a written quantitative food frequency questionnaire (FFQ) which was validated for pregnant women in Brazil [32].

Based on women´s pre-pregnancy weight and measured height, we classified them into one of four pre-pregnancy body mass index (BMI) categories (<18.5, 18.5–24.9, 25.0–29.9, or ≥30.0 kg/m^2^) [33]. According to weight gain at the time of recruitment (weight measured by the researcher minus the self-reported pre-pregnancy weight), we also classified participants into one of three gestational weight gain categories (insufficient, adequate, or excessive) according to the recommendations of the Institute of Medicine [33].

The FFQ assessed food consumption in the last six months. To facilitate the quantification of portions and to reduce recall bias, we showed participants photos of standard serving tools (cups, plates, spoons), as well as photos of different food portion sizes. Using the dietary guidelines proposed for the Brazilian population, we converted portion sizes to grams or milliliters [34]. Portion sizes were categorized as small, medium, large, and extra large using the 25th, 50th, 75th, and 100th percentiles, respectively. We then calculated the distribution of the reported food consumption of the past six months via assessing the daily, weekly, and monthly food frequency [32]. Participant´s macro and micronutrient intake were calculated using the Dietpro Clinic nutrition software (AS Systems, 2019, version 6.1, Viçosa, Brazil). To assess the adequacy of participants´ food consumption, we followed the dietary reference intake (DRI 2002) recommendations for pregnant women [35]. If the frequency was between the estimated average need (average reference value) and the upper tolerable limit of intake, food intake was categorized as adequate.

### 2.4. Sample Size

We used the first 60 eligible pregnant women with GDM (convenience sample). The 60 participants (30 with hepatic steatosis and 30 with a normal liver US) were matched for self-reported skin color (brown versus other), pre-pregnancy BMI category (<18.5 versus 18.5–24.9 versus ≥25 Kg/m^2^), and for age (± 2 years) [29].

### 2.5. Statistical Analysis

We used the Student’s *t* test or Fisher’s exact test to compare participant characteristics in the two groups. We present the median (1st–3rd quartile) macronutrient and micronutrient intake of participants in each group. Differences in dietary intakes between the two groups were assessed using the Mann–Whitney test. *p* values < 0.05 were considered statistically significant.

Multivariate logistic regression was performed to examine the relationship between energy, macro, and micronutrient intake and the number of abnormal values on the GTT. For this analysis, consumption for each of the dietary items was categorized in tertiles (T1: low, T2: average, T3: high). Each of the dietary items was analyzed separately to estimate the crude odds ratios (ORs) and the 95% confidence intervals (CIs) for the likelihood of having 2–3 abnormal glucose values on the GTT. An adjusted model was created to adjust for the following covariates: age, race, income, and education.

We used the software SPSS version 19.0 (IBM Corp., Armonk, NY, USA) for all analyses.

## 3. Results

Most (51.7%, *n* = 31) of the participants had only 1 abnormal GTT value. The participant characteristics did not differ significantly between the two groups (Table 1).

Table 2 presents the participants’ dietary pattern over the last six months. Participants in both groups had an average dietary intake of fiber, vitamin A, and vitamin E below the recommended level, and a higher than recommended protein intake. There were no significant differences between groups in caloric, macronutrient, and vitamin A, E, selenium, and zinc intake. The average daily consumption of fiber (11.9 vs. 11.0 g, *p* = 0.049), vitamin D (40.6 vs. 40.4 mcg, *p* = 0.049), and vitamin C (180.0 vs. 151.0 mg, *p* = 0.008) was significantly higher in the group with 1 abnormal GTT value than in the group with 2–3 abnormal GTT values.

Table 3 presents the risk of having 2–3 abnormal GTT values according to tertiles of dietary items consumed. The likelihood of having 2–3 abnormal values decreased significantly as the consumption of fiber increased in tertiles. When compared with the lowest tertile, higher fiber consumption was associated with an 80% lower likelihood of having 2–3 abnormal GTT values (highest versus lowest tertile: aOR 0.20, 95% CI 0.04 to 0.95, *p* = 0.043). Likewise, higher vitamin D consumption was associated with an 81% lower likelihood of having 2–3 abnormal GTT values (highest versus lowest tertile: aOR 0.19, 95% CI 0.04 to 0.90, *p* = 0.036). Similarly, when compared to women with the lowest ingestion, those showing an average ingestion of vitamin C had an 83% lower likelihood of having 2–3 abnormal GTT values (second versus first tertile: aOR 0.17, 95% CI 0.03 to 0.82, *p* = 0.028), while women showing the highest ingestion of vitamin C had a 92% lower likelihood of having 2–3 abnormal GTT values (third versus first tertile: aOR 0.08, 95% CI 0.01 to 0.51, *p* = 0.007).

## 4. Discussion

We found significant differences between the food intake of women with 1 abnormal GTT value and those with 2–3 abnormal values. There were no significant differences in the consumption of macronutrients between the two groups. However, the consumption of fibers, as well as vitamins C and D were significantly higher in women with 1 abnormal value than in those with 2–3 abnormal values. The likelihood of having 2–3 abnormal GTT values decreased significantly as the consumption of fibers, vitamin C, and vitamin D increased.

GDM is a disease that involves genetic factors, lifestyle, and eating habits [36,37,38]. Several studies confirm the role of diet in the development of GDM. Women with diets which are rich in ultra-processed foods, low in fibers, and rich in simple carbohydrates associated with the consumption of sugary drinks have a higher risk of developing GDM [39,40,41,42,43]. Our study advances the knowledge in this field by showing an inverse relationship between the consumption of fibers, and of vitamins C and D, and the number of abnormal glucose values on the GTT.

Our finding of a lower fiber intake in the group with 2–3 abnormal GTT values, a possibly more severe subtype of GDM [13,14,15,16], is consistent with the literature. Xu et al. showed that the intake of total fiber and fruit fiber in the first and second trimester of pregnancy was significantly correlated with a decreased risk of GDM [44]. A meta-analysis of 8 trials involving 752 patients with GDM concluded that additional fortification with dietary fiber supplements significantly reduced fasting glucose, 2 h postprandial glucose, and the glycated hemoglobin levels in the study participants [45]. Dietary fiber plays an important role in controlling postprandial blood glucose because it delays gastric emptying, blood glucose levels, insulin resistance, and the metabolic profile of healthy and diabetic individuals [46,47].

There is evidence that a diet which is rich in antioxidants could reduce the incidence of GDM [48]. Normal pregnancy is characterized by increased levels of free radicals and lipid peroxides [49]. However, hyperglycemia during pregnancy is associated with increased oxidative stress, which impairs the insulin-dependent glucose uptake and increases apoptosis and placental dysfunction. These factors can contribute to a greater pro-inflammatory state which, in turn, may worsen hyperglycemia and the severity of GDM [50,51].

Our findings of lower vitamin C and D values in women with 2–3 abnormal GTT values align with those reported in the literature, showing that adequate vitamin D and C intake can have a protective role in the development GDM and may improve glycemic control [52,53,54,55,56]. In a meta-analysis involving 15 observational studies with over 8000 participants, Zhou et al. showed that, among women with low vitamin C ingestion, the risk of developing GDM was 2.72-fold higher than in those with higher vitamin C ingestion [55]. The beneficial effect of vitamin C on glucose metabolism is probably due to its anti-inflammatory and antioxidant activities, which could help to compensate for the increased oxidative stress and insulin resistance which are typical of pregnancy. On the other hand, vitamin D affects glucose metabolism by increasing insulin sensitivity, and women with low levels of vitamin D have a higher risk of developing GDM [53].

This is the first study that investigated the association between usual food consumption and the number of abnormal glucose values on the GTT. This approach is innovative and aligns with the recent concept that GDM is a disease with different phenotypes [57]. Several investigators suggest the existence of different metabolic subtypes of GDM, possibly associated with better or worse maternal/perinatal outcomes, which could be identified based on GTT results [14,15,16,58,59,60,61,62]. The study has several limitations. Due to its small sample size, the study may have been underpowered to detect other associations between dietary patterns and the number of abnormal GTT values. Moreover, the study design and the absence of a control group (pregnant women with a normal GTT) prevent inferring a causal relation between dietary intake and GTT results. Another study limitation was that, since we used only one FFQ for the six months that preceded the diagnosis of GDM, we cannot exclude recall bias.

Our study does not conclusively demonstrate an association between the intake of the various nutrients studied and the number of abnormal values on the GTT. More studies are needed to confirm these findings. Future studies should involve a larger number of participants, a more diverse group of women with GDM, and a control group of pregnant women with normal GTT results.

## 5. Conclusions

Our results suggest a possible association between the consumption of dietary fiber and some antioxidant nutrients (vitamin D and C) and the number of abnormal glucose values on the GTT at the time of GDM diagnosis. As hypothesized, women with a larger number of abnormal glucose values on the GTT had diets that were poorer in antioxidants and fibers. These findings contribute to the evolving field of research concerning the role of diet in the development of different subtypes of GDM.

## Figures and Tables

**Table 1:** Characteristics of 60 pregnant women with gestational diabetes mellitus according to the glucose tolerance test results.

Variables	Number of Abnormal Glucose Values on GTT *		*p* Value ^#^
	1 (*n* = 31)	2–3 (*n* = 29)	
Age, years, mean (SD)	33.0 (5.7)	34.5 (6.1)	0.337
Gestational age, weeks, mean (SD)	28.0 (2.9)	27.2 (2.6)	0.268
Skin color			0.146
Brown	11 (35.5)	10 (34.5)	
White	2 (6.5)	7 (24.1)	
Black	18 (58.1)	12 (41.4)	
Marital status			0.782
Married	10 (32.3)	8 (27.6)	
Single	21 (67.7)	21 (72.4)	
Education, years			0.140
0–7	2 (6.5)	6 (20.7)	
≥8	29 (93.5)	23 (79.3)	
Monthly family income			0.208
<256 USD	17 (54.8)	11 (37.9)	
≥256 USD	14 (45.2)	18 (62.1)	
Religion			0.177
Catholic	18 (58.1)	22 (75.9)	
Other	13 (41.9)	7 (24.1)	
Family history of diabetes			0.800
Yes	17 (54.8)	17 (58.6)	
No	14 (45.2)	12 (41.4)	
Chronic hypertension			0.229
No	31 (100.0)	27 (93.1)	
Yes	0 (0.0)	2 (6.9)	
Physically active			0.783
No	22 (71.0)	19 (65.5)	
Yes	9 (29.0)	10 (34.5)	
Pre-pregnancy BMI, kg/m^2^			0.398
<18.5	0 (0.0)	1 (3.4)	
18.5–24.9	3 (9.7)	2 (6.9)	
25.0–29.9	10 (32.3)	14 (48.3)	
≥30.0	18 (58.1)	12 (41.4)	
Gestational weight gain			1.000
Insufficient	14 (45.2)	13 (44.8)	
Adequate	5 (16.1)	5 (17.2)	
Excessive	12 (38.7)	11 (37.9)	
Hepatic steatosis			0.930
Absent	16 (51.6)	14 (48.3)	
Mild	12 (38.7)	13 (44.8)	
Moderate	3 (9.7)	2 (6.9)	
Abnormal glucose value in GTT * ^EUR^			0.945
Fasting	19 (61.3)	21 (72.4)	
1 h	5 (16.1)	19 (65.5)	
2 h	7 (22.6)	27 (93.1)	

BMI: Body mass index, GTT: Glucose tolerance test, SD: Standard deviation, USD: US dollars. All values represent numbers (%) unless otherwise noted. * 75 g GTT cutoffs for GDM diagnosis: fasting 92–125, 1 h ≥ 180, 2 h 153–199 mg/dL. ^#^ Student’s *t* test or Fisher’s exact test. ^EUR^ The total number in the group with 2–3 abnormal glucose values is > total group number (*n* = 29) because each participant had 2 or more abnormal glucose values.

**Table 2:** Food consumption of 60 pregnant women with gestational diabetes according to the number of abnormal values on the glucose tolerance test.

Variable	Nutritional Recommendation *	Number of Abnormal Glucose Values on GTT **		*p* Value ^#^
		1 (*n* = 31)	2–3 (*n* = 29)	
Calories	1800–2500	2000.5 (1779.0–2230.0)	1905.0 (1696.0–2111.0)	0.105
Carbohydrate	45–65%	57.9 (50.0–63.5)	56.4 (53.5–63.9)	0.676
Protein	10–15%	15.8 (14.3–18.5)	16.1 (13.5–16.9)	0.245
Lipid	25–30%	27.5 (22.4–32.1)	27.1 (23.1–30.6)	0.471
Dietary fiber	28 g	11.9 (10.8–16.2)	11.0 (9.1–14.2)	0.049
Vitamin A	550–3000 μg	144.0 (68.7–298.0)	146.0 (77.1–199.0)	0.270
Vitamin D	50 µg	40.6 (40.3–80.2)	40.4 (20.8–40.7)	0.049
Vitamin E	12–1000 mg	6.0 (4.5–8.3)	6.0 (4.6–8.1)	0.427
Vitamin C	70–2000 mg	180.0 (157.0–275.0)	151.0 (117.0–204.0)	0.008
Selenium	49–400 μg	43.5 (32.0–47.1)	39.0 (32.2–49.2)	0.350
Zinc	9.5–40 mg	5.6 (4.2–6.9)	5.2 (4.1–6.0)	0.185

GTT: Glucose tolerance test. All values represent median (1st–3rd quartile). * Estimated average needs to assess the adequacy of intake of pregnant women based on the recommended dietary intake and maximum tolerable intake. ** 75 g GTT cutoffs for GDM diagnosis: fasting 92–125, 1 h ≥ 180, 2 h 153–199 mg/dL. ^#^ Mann–Whitney test.

**Table 3:** Likelihood of having 2–3 abnormal glucose values on the GTT according to tertiles of nutrient intake.

Nutrients	2–3 Abnormal Values (*n* = 29)	1 Abnormal Value (*n* = 31)	OR	95% CI	*p* Value	aOR	95%CI	*p* Value
Calories								
T1: <1809.4	11 (37.9%)	9 (29.0%)	1.00			1.00		
T2: 1809.4–2128.9	11 (37.9%)	9 (29.0%)	1.00	0.29; 3.48	1.000	1.01	0.25; 4.02	0.996
T3: ≥2129.0	7 (24.1%)	13 (41.9)	0.44	0.12; 1.57	0.207	0.54	0.13; 2.28	0.400
Carbohydrate								
T1: <255.1	12 (41.4%)	8 (25.8)	1.00			1.00		
T2: 255.1–297.1	9 (31.0%)	11 (35.5%)	0.54	0.15; 1.91	0.344	0.27	0.05; 1.25	0.095
T3: ≥297.2	8 (27.6%)	12 (38.7%)	0.44	0.12; 1.57	0.209	0.40	0.09; 1.74	0.225
Protein								
T1: <68.4	13 (44.8%)	7 (22.6%)	1.00			1.00		
T2: 68.4–87.6	9 (31.0%)	11 (35.5%)	0.44	0.12; 1.57	0.207	0.50	0.10; 2.28	0.371
T3 ≥87.7	7 (24.1%)	13 (49.1%)	0.29	0.07; 1.06	0.062	0.34	0.08; 1.43	0.143
Lipid								
T1: <52.1	12 (41.4%)	8 (25.8%)	1.00			1.00		
T2: 52.1–64.6	10 (34.5%)	10 (32.3%)	0.66	0.19; 2.33	0.526	0.84	0.20; 3.39	0.807
T3: ≥64.7	7 (24.1%)	13 (41.9%)	0.35	0.09; 1.29	0.117	0.67	0.14; 3.11	0.616
Dietary fiber								
T1: <10.6	13 (44.8%)	7 (22.6%)	1.00			1.00		
T2: 10.6–13.9	8 (27.6%)	12 (38.7%)	0.36	0.10; 1.29	0.117	0.33	0.07; 1.48	0.150
T3: ≥14.0	8 (27.6%)	12 (38.7%)	0.35	0.09; 1.29	0.117	0.20	0.04; 0.95	0.043
Vitamin A								
T1: <79.6	10 (34.5%)	10 (32.3%)	1.00			1.00		
T2: 79.6–199.0	12 (41.4%)	8 (25.8%)	1.50	0.42; 5.25	0.526	1.03	0.23; 4.60	0.960
T3: ≥199.1	7 (24.1%)	13 (41.9%)	0.53	0.15; 1.92	0.339	0.54	0.13; 2.13	0.384
Vitamin D								
T1: <40.3	13 (44.8%)	7 (22.6%)	1.00			1.00		
T2: 40.3–40.9	9 (31.0%)	11 (35.5%)	0.44	0.12; 1.57	0.207	0.38	0.10; 1.76	0.219
T3: ≥41.0	7 (24.1%)	13 (41.9%)	0.29	0.79; 1.06	0.062	0.19	0.04; 0.90	0.036
Vitamin C								
T1: <145.1	14 (41.3%)	6 (19.4%)	1.00			1.00		
T2: 145.1–204.0	8 (27.6%)	12 (38.7%)	0.28	0.07; 1.05	0.061	0.17	0.03; 0.82	0.028
T3: ≥204.1	7 (24.1%)	13 (41.9%)	0.23	0.06; 0.86	0.030	0.08	0.01; 0.51	0.007
Vitamin E								
T1: <4.9	10 (34.5%)	10 (32.3%)	1.00			1.00		
T2: 4.9–7.8	10 (34.5%)	11 (35.5%)	0.91	0.26; 3.10	0.879	0.97	0.23; 4.03	0.975
T3: ≥7.9	9 (31.0%)	10 (32.3%)	0.90	0.25; 3.16	0.869	0.86	0.20; 3.63	0.843
Selenium								
T1: <40.0	11 (37.9%)	9 (29.9%)	1.00			1.00		
T2: 40.0–45.6	8 (27.6%)	12 (38.7%)	0.54	0.15; 1.91	0.344	0.78	0.18; 3.25	0.735
T3: ≥45.7	10 (34.5%)	10 (32.3%)	0.81	0.23; 2.84	0.752	0.84	0.21; 3.34	0.810
Zinc								
T1: <4.7	9 (31.0%)	11 (35.5%)	1.00			1.00		
T2: 4.7–5.9	11 (37.9%)	9 (29.0%)	1.49	0.43; 5.19	0.528	2.09	0.44; 9.79	0.349
T3: ≥6.0	9 (31.0%)	11 (35.5%)	1.00	0.28; 3.48	1.000	0.92	0.22; 3.85	0.912

CI: Confidence interval. GTT: Glucose tolerance test. OR: Odds ratio. aOR: adjusted OR (age, race, income, and education). T: tertile.

## Data Availability

The data presented in this study are available on request from the corresponding author.
